# Correlation Between C-Reactive Protein and Lipid Analytes in Dry Age-Related Macular Degeneration: A Retrospective Study

**DOI:** 10.7759/cureus.51935

**Published:** 2024-01-09

**Authors:** Naif S Sannan, Mohieldin Elsayid, Ghadi Alsharif, Majed Ramadan, Amani Y Alhalwani, Rowaid M Qahwaji, Ahmad Arbaeen, Waseem A Aalam, Abdullah S Alqahtani, Karim Talat

**Affiliations:** 1 Department of Clinical Laboratory Sciences, King Saud Bin Abdulaziz University for Health Sciences, Jeddah, SAU; 2 Department of Biomedical Research, King Abdullah International Medical Research Center, Jeddah, SAU; 3 Department of Clinical Laboratory, King Abdulaziz University, Jeddah, SAU; 4 Department of Laboratory Medicine, Faculty of Applied Medical Sciences, Umm Al-Qura University, Makkah, SAU; 5 Department of Ophthalmology, College of Medicine, University of Jeddah, Jeddah, SAU; 6 Department of Ophthalmology, King Abdulaziz Medical City, Ministry of National Guard - Health Affairs, Jeddah, SAU

**Keywords:** serum biomarkers, crp, lipid profile, inflammation, dry age-related macular degeneration

## Abstract

Introduction: To date few studies have investigated the correlation between inflammatory markers and lipoproteins in the serum of age-related macular degeneration (AMD) patients, often reporting conflicting findings. This study aimed to investigate the correlation between lipid analytes and C-reactive protein (CRP) levels in individuals diagnosed with dry AMD.

Methods: A standard clinical lipid panel (total cholesterol, triglycerides, high-density lipoprotein [HDL], and low-density lipoproteins) and CRP laboratory results were retrospectively collected from the medical records of patients with dry AMD and age- and sex-matched controls.

Results: The study included 90 patients with dry AMD and 270 patients without AMD. In univariate analysis, CRP showed a higher mean value in cases than in controls. After adjusting for age and sex, CRP and triglyceride levels showed significant differences between cases and controls. Pearson’s correlation analysis revealed a significant negative correlation between CRP and HDL levels in the dry AMD group (n=90). Other lipid analytes showed no significant correlations with CRP.

Conclusion: Our findings add to the growing body of evidence linking inflammation to AMD. Although it is unclear whether changes in serum CRP and triglyceride levels are the causes or effects, monitoring both analytes may be beneficial as an early disease predictor, especially in individuals with a family history of AMD. The negative correlation between CRP and HDL (i.e., inflammation and good cholesterol) may be targeted for future therapies.

## Introduction

The global prevalence of age-related macular degeneration (AMD) is projected to increase by 40%, from 196 million in 2020 to 288 million by 2040 [1]. AMD is caused by the buildup of drusen in the macula, leading to vision impairment and/or blindness [2]. AMD is a multifactorial condition involving complex interactions between causes and risk factors, and its pathogenesis is associated with inflammation, oxidative stress, altered cholesterol metabolism, impaired retinal pigment epithelium, and defects in lipid pathway genes [3-7]. Age, elevated C-reactive protein (CRP) levels, and abnormal lipid analyte levels are risk factors for the development of AMD [8].

Many studies have analyzed serum lipoprotein profiles in patients with AMD and compared these with profiles of non-AMD controls; however, no clear consensus has been reported. In particular, the AMD Risk Factors Study Group [9], the Beaver Dam Eye Study [10], and the Rotterdam AMD Study [11] reported a correlation between high serum high-density lipoprotein (HDL) cholesterol levels and AMD. The Rotterdam AMD Study also showed that high HDL levels were associated with an elevated risk of AMD, independent of the apolipoprotein E genotype [11]. Other studies have reported elevated levels of total cholesterol, triglycerides, and low-density lipoproteins (LDL) [12,13], or higher levels of HDL and low triglycerides in those with AMD [14]. In contrast, Wang et al. [15] showed that elevated serum total cholesterol, LDL, and triglyceride levels were associated with a lower risk of developing AMD, whereas other studies reported no significant correlation between serum lipoprotein levels and AMD [13,16]. Such inconsistencies may result from different study designs, insufficient sample size for statistical power, variations in case-control definitions and disease mechanisms among participants, or other uncontrolled confounders.

CRP is a highly conserved protein released by the liver in response to inflammatory cytokines and is often used as a nonspecific marker of systemic inflammation; its concentration increases in circulation during inflammatory illnesses, such as some autoimmune diseases, some cardiovascular diseases, and infections [17]. Seddon et al. [18] reported elevated CRP levels in patients with AMD, independent of other risk factors, such as smoking and obesity. While the direct impact of elevated CRP levels on AMD remains unclear, recent studies suggest a potential association with choroidal thinning [19] and an elevated risk of disease progression [20].

Information on the relationship between CRP and lipoproteins in AMD is limited in the literature, often with conflicting results reported. Therefore, this study aimed to examine the association between serum CRP, total cholesterol, triglycerides, HDL, and LDL levels in Saudi patients with dry AMD.

## Materials and methods

This study involved a secondary analysis of previously reported patient data [21,22]. In summary, we conducted a review of electronic medical records at King Abdulaziz Medical City in Jeddah, Saudi Arabia, to identify new patients with AMD who were 50 years of age or older and exhibited macular drusen in at least one eye, with or without signs of geographic atrophy, along with other fundus characteristics. Exclusion criteria encompassed individuals with wet AMD in their other eye, inflammatory ocular conditions, different ophthalmic disorders, malignancies, autoimmune diseases, high leukocyte counts (>11,000 cells/mm^3^), low leukocyte counts (<4,000 cells/mm^3^), high platelet counts (>450,000 cells/mm^3^), and low platelet counts (<150,000 cells/mm^3^). Patients receiving lipid-lowering drugs were also excluded from the study. Lipid profiles and CRP results were retrospectively collected for the AMD cases and randomly selected age- and sex-matched cataract controls without AMD [laboratory data was generated by the Abbott Architect chemistry analyzer (Abbott Diagnostics, Chicago, IL, USA)]. The ages of the individuals in the control group were limited to being within five years of the mean age of the individuals in the case group.

For the sample size calculation, a matched case-control study was performed (the case-control ratio was 1:3). Differences between the case and control groups were investigated using multivariate analysis adjusted for age and sex. A chi-square test for categorical data and a t-test for numeric variables were used to investigate the relationships between demographics and serum results among cases and controls in the univariate analysis. Due to the nature of our findings, we used an analysis of covariance (ANCOVA) to evaluate the models. All models met the assumptions of a linear relationship between the dependent variable and the covariate, as well as the homogeneity of the regression slopes. All confidence intervals were set at 95%, and the p-values were two-sided. SAS (version 9.4, SAS Institute Inc., Cary, NC, USA) was used for all the analyses. Data are presented as numbers (%) or as mean ± standard deviation. A p-value ≤0.05 was considered statistically significant.

Ethical approval was obtained from the Institutional Review Board of King Abdullah International Medical Research Center (Riyadh, Saudi Arabia), and the study was conducted in accordance with the principles of the Declaration of Helsinki (study number #SP21J/083/03&RJ20/106/J). Informed consent was obtained from all study participants, and the study protocol was approved by the institutional review board.

## Results

After applying the inclusion and exclusion criteria, the lipid profiles and CRP levels of 90 patients with dry AMD [41 (45.5%) males and 49 (42.5%) females] and 270 control individuals without AMD [115 (42.5%) males and 155 (57.4%) females] were analyzed. The mean ages were 71 ± 9 and 70 ± 7 years for the cases and the controls, respectively. In the univariate analysis (Table 1), the mean CRP level was higher in the cases than in the controls (3.15 and 2.07 mg/l, respectively; p = 0.008). No significant differences were noted in total cholesterol, HDL, LDL, or triglyceride levels between the cases and the controls.

**Table 1:** Baseline demographic and clinical characteristics of dry AMD cases and controls without AMD using univariate analysis ^1^Chi-square test for categorical variables, and t-test for numeric variables. ^*^Significant p value ≤0.05. “n”, sample size; %, percentage; HDL, high-density lipoprotein cholesterol; LDL, low-density lipoprotein cholesterol; CRP, C-reactive protein; AMD, age-related macular degeneration.

	Cases of dry AMD patients, n (%)	Controls without AMD, n (%)	p-Value, cases vs controls^1^
Total	90 (22.55)	270 (77.44)	
Age, years, mean ± SD	71 ± 9	70 ± 7	0.27
Sex			0.63
Male	41 (45.56)	115 (42.59)	
Female	49 (54.44)	155 (57.41)	
Total cholesterol (mmol/l), mean (CI)	4.3 (4.1, 4.5)	4.6 (4.3, 4.6)	0.15
HDL (mmol/l), mean (CI)	1.11 (1.05, 1.15)	1.08 (1.06, 1.11)	0.35
LDL (mmol/l), mean (CI)	2.68 (2.47, 2.88)	2.81 (2.68, 2.92)	0.22
Triglycerides (mmol/l), mean (CI)	1.32 (1.12, 1.35)	1.48 (1.4, 1.56)	0.31
CRP (mg/l), mean (CI)	3.15 (1.66, 4.64)	2.07 (1.68, 2.47)	0.008^*^

After adjusting for age and sex (Table 2), the CRP and triglyceride levels showed significant differences (p = 0.006 and p = 0.05, respectively) between the cases and the controls. The dry AMD group had higher CRP and lower triglyceride levels than the control group.

**Table 2:** Regression analysis for predicting factors associated with dry AMD in cases vs controls without AMD (adjusted for age and sex) ^1^ANCOVA was used to estimate differences in covariances across patients with dry AMD and without AMD, adjusted for age and sex, and the Kruskal-Wallis test for non-normal numeric variables. ^*^Significant p value ≤0.05. HDL, high-density lipoprotein cholesterol; LDL, low-density lipoprotein cholesterol; CRP, C-reactive protein; AMD, age-related macular degeneration; ANCOVA, analysis of covariance.

Analyte	Beta coefficient (β)	Standard error (SE)	p-Value, cases vs controls^1^
Total cholesterol	0.13	0.11	0.22
HDL	-0.011	0.025	0.65
LDL	0.06	0.04	0.14
Triglycerides	0.09	0.05	0.05*
CRP	19.88	7.29	0.006*

The Pearson’s correlation coefficients of CRP levels with HDL, LDL, and triglyceride levels were -0.24 (p = 0.05), 0.11 (p = 0.37), and 0.019 (p = 0.88), respectively. The correlation coefficient of CRP with HDL was significant (-0.24, p = 0.05).

## Discussion

The main aim of this study was to investigate the associations among systemic CRP, total cholesterol, triglycerides, HDL, and LDL levels in patients diagnosed with dry AMD. We focused entirely on dry AMD to better understand early disease processes. To the best of our knowledge, this is the first study to quantitatively assess CRP levels and lipid analytes in Saudi patients with AMD. A better understanding of CRP and lipid levels is essential for developing diagnostic and therapeutic strategies for AMD.

Spectroscopic measurements have demonstrated the presence of lipoproteins in Bruch’s membrane, with a peak density of systemic LDL, HDL, and very-low-density lipoproteins [23]. Lipids and lipoproteins are implicated in the production of extracellular lesions in the aging Bruch’s membrane, basal deposits, and drusen (localized deposits of extracellular debris made up of lipids). Several studies have examined the relationship between lipid profiles and the risk of developing AMD [10-15,24]. Furthermore, several epidemiological studies have reported a substantial association between HDL levels and an increased risk of soft drusen, neovascular AMD, and geographic atrophy [25,26]. However, it is still unclear whether systemic lipids directly affect AMD or recapitulate lipid metabolism in the retina.

In this study, the levels of HDL and other lipid analytes were compared between cases and controls, and after statistical adjustment for the effects of age and sex, triglyceride levels were found to be significantly lower in the AMD group than in the control group. Similar triglyceride findings have been reported in participants from the European Eye Epidemiology Consortium and the Rotterdam Study [14]. Our results may indicate a similar disease mechanism in the Saudi population. In addition, our study did not find significant variations in the total cholesterol, HDL, and LDL levels between cases and controls, unlike those noted in another report on neovascular AMD [14], suggesting different pathogenesis with different systemic processes.

Elevated systemic CRP levels may reflect the inflammatory macular microenvironment in dry AMD tissues. To the best of our knowledge, Seddon et al. were the first to investigate the association between high CRP levels and AMD development; in the Age-Related Eye Disease Study cohort, they recorded increased CRP levels in individuals with intermediate and advanced AMD compared with the levels found in normal controls [18]. Seddon et al. also found that higher baseline CRP levels were associated with the progression of intermediate AMD to advanced stages [20]. Mitta et al. reported that elevated CRP levels could predict the future risk of AMD [27]. Similarly, we found that both triglyceride and CRP levels were altered in AMD cases compared to controls, which substantiates the findings of previous work by Wu et al., who reported that the inflammatory status of the patient plus the apolipoprotein E genotype could modulate triglyceride levels [28].

The inverse correlation between CRP and HDL has been previously reported in patients with atherosclerosis [29], non-small-cell lung carcinoma [30], cardiovascular disease treated with lipid-modifying therapies [31], and AMD [32]. HDL has putative pleiotropic anti-inflammatory characteristics [33], and reduced HDL levels may result in a decline in HDL-anti-inflammatory function, which may contribute to AMD development. This combination of CRP-HDL levels may have powerful predictive value for early disease detection and may be a potential target for future therapies. The clinical value of these analytes requires further investigation in clinical trials.

This study has some limitations. First, the inclusion criteria allowed the cases and controls to potentially have one or more prevalent and age-related conditions (e.g., coronary heart disease, stroke, peripheral artery disease, diabetes, and hypertension) to attain a sufficient sample size for this aging population in the study. Second, we studied blood lipoproteins and CRP levels, which could differ from those circulating locally in the eye. Furthermore, due to the retrospective nature of this study, no adjustments were made for AMD risk variables that could directly implicate CRP and triglyceride levels, such as smoking, body mass index, and supplement intake. Several mechanisms could lead to inflammatory responses in patients with AMD, including mechanisms involving oxidative stress induced by established risk factors, such as smoking [6,34], low intake of dietary antioxidants [35], high consumption of dietary fat [36], and obesity [37]. Finally, cardiovascular diseases and related risk factors, such as cigarette smoking, obesity, and hypertension, are associated with the development and progression of AMD [34,37,38]. The mechanism of AMD drusen formation is similar to that of atherosclerotic plaque formation [39]. In future studies, adopting prospective designs and refining inclusion criteria to focus on individuals without prevalent age-related conditions would enhance conclusions about the correlation between blood lipoproteins, CRP levels, and AMD. Additionally, incorporating comprehensive adjustments for relevant AMD risk variables, such as genetics, smoking, body mass index, and supplement intake, would further enhance the study's overall conclusions.

## Conclusions

The inverse correlation of CRP-HDL may shed more light on the underlying disease mechanisms and could be of clinical utility, including identifying individuals with a higher risk of developing AMD who may benefit from routine eye examinations, adherence to lifestyle recommendations, and treatment protocols. Further large-scale and multicenter prospective studies are needed to generalize these findings.
